# A Novel Picornavirus Discovered in White Leg Shrimp *Penaeus vannamei*

**DOI:** 10.3390/v13122381

**Published:** 2021-11-28

**Authors:** Shuang Liu, Tingting Xu, Chong Wang, Tianchang Jia, Qingli Zhang

**Affiliations:** 1 Qingdao Key Laboratory of Mariculture Epidemiology and Biosecurity, Key Laboratory of Maricultural Organism Disease Control, Ministry of Agriculture, Yellow Sea Fisheries Research Institute, Chinese Academy of Fishery Sciences, Qingdao 266071, China; liushuang@ysfri.ac.cn (S.L.); xutingting83@163.com (T.X.); wangchongyilin@163.com (C.W.); jiatc2021@163.com (T.J.); 2 Laboratory for Marine Fisheries Science and Food Production Processes, Pilot National Laboratory for Marine Science and Technology, Qingdao 266071, China

**Keywords:** *Penaeus vannamei* picorna viruses (*Pv*PV), *Picornavirales*, *Dicistroviridae*, *Penaeus vannamei*, shrimp

## Abstract

Global shrimp farming is increasingly threatened by various emerging viruses. In the present study, a novel picornavirus, *Penaeus vannamei* picornavirus (*Pv*PV), was discovered in moribund White leg shrimp (*Penaeus vannamei*) collected from farm ponds in China in 2015. Similar to most picornaviruses, *Pv*PV is non-enveloped RNA virus, with a particle diameter of approximately 30 nm. The sequence of the positive single-stranded RNA genome with a length of 10,550 nts was characterized by using genome sequencing and reverse transcription PCR. The existence of *Pv*PV related proteins was further proved by confirmation of viral amino acid sequences, using mass spectrometry analysis. Phylogenetic analysis based on the full-length genomic sequence revealed that *Pv*PV was more closely related to the Wenzhou shrimp virus 8 than to any other dicistroviruses in the order *Picornavirales*. Genomic sequence conservative domain prediction analysis showed that the *Pv*PV genome encoded a large tegument protein UL36, which was unique among the known dicistroviruses and different from other dicistroviruses. According to these molecular features, we proposed that *Pv*PV is a new species in the family *Dicistroviridae*. This study reported the first whole-genome sequence of a novel and distinct picornavirus in crustaceans, *Pv*PV, and suggests that further studies of *Pv*PV would be helpful in understanding its evolution and potential pathogenicity, as well as in developing diagnostic techniques.

## 1. Introduction

Viral diseases of *Penaeus vannamei* are important restrictive considerations in the shrimp farming industry in the world [1,2]. Major *P. vannamei* viruses impacting global shrimp farming to date have included white spot syndrome virus (WSSV) [3,4], covert mortality nodavirus (CMNV) [5], infectious hypodermal and hematopoietic necrosis virus (IHHNV) [6], Taura syndrome virus (TSV) [7], yellow head disease virus (YHV) [8], infectious myonecrosis virus (IMNV) [9] and Decapod iridescent virus 1 (DIV1). Recently, global shrimp farming has been increasingly threatened by emerging viruses, and the diseased shrimp individuals are often infected by more than one species of virus [10,11,12]. The genomes of the major shrimp viruses have now been fully sequenced, and the viruses with published genomes include WSSV [13,14], CMNV [15], IHHNV [16], TSV [17], YHV [18], shrimp hepatopancreatic parvovirus (HPV) [19,20], *Penaeus vannamei* nodavirus (*Pv*NV) [21] and IMNV [22]. Further studies on the genome sequence of shrimp viruses would be helpful in relation to their evolution, diagnostic techniques and control strategies.

The order *Picornavirales* has undergone a significant expansion in recent years, due principally to the identification of previously unknown picornaviruses discovered in vertebrates, arthropods, algae, humans, insects and plants using “next-generation” sequencing [23]. The members of *Picornavirales* have non-enveloped icosahedral virions about 30 nm in diameter, with a pseudo-T = 3 (abbreviated “p = 3”) structure [24]. The existence of the Hel–Pro–Pol core replicative module, including the “Hel” domain (a superfamily III helicase), “Pro” domain (a chymotrypsin-like proteinase) and “Pol” domain (a super-family I RNA-dependent RNA polymerase (RdRp)), has proved to be a primary feature of picornavirus genomes [25,26,27,28,29]. The CP module encoded upstream of the Hel–Pro–Pol module uniquely discriminates the members of the order *Picornavirales* from other picorna-like viruses [24]. Moreover, some virions of the viruses in the family *Picornaviridae* additionally include a small protein, VP4, encoded upstream of the CP module in the polyprotein [30,31]. The diversity of genomic structure indicates the ever-increasing genetic diversity of picornaviruses. However, the biological significance of many newly discovered picornaviruses is still unknown.

Taura syndrome virus (TSV) was the first characterized picorna virus infecting an invertebrate, *P. vannamei,* other than an insect [32]. Taura syndrome caused by TSV is one of the major diseases in penaeid shrimp and has impacted the shrimp farming industry in the past decade in some shrimp farming countries [33]. The main host of TSV was *P. vannamei;* in addition, it has also been reported to infect other penaeid shrimp species [34]. TSV primarily affected *P. vannamei* and *P. stylirostris* in the Americas at the beginning and then spread to farming of *P. vannamei* in Southeast Asia, where it has been responsible for acute mortality levels among farmed penaeid shrimp in Taiwan [35,36].

In this paper, we describe the genome sequence, morphological features and phylogenetic relationships of a novel picorna virus discovered in *P. vannamei* sampled from a farm in China in 2015. Based on the evidence obtained in the present study supporting a new virus species, we suggest a name, *P. vannamei* picornavirus (*Pv*PV), for this virus.

## 2. Materials and Methods

### 2.1. Shrimp Samples and Ethics Statement

On 15 July 2015, diseased juvenile *P. vannamei* individuals (body length 5–7 cm, NO. 20150715007) with the same disease syndrome were sampled from a shrimp farm pond in Weifang, Shandong Province, China. The hepatopancreas tissues of the sampled individuals were fixed in 2.5% glutaraldehyde solution (Sinopharm, Beijing, China) for transmission electron microscope observation, and the remaining parts of the diseased shrimp sample NO. 20150715007 were preserved at −80 °C for viral isolation and meta-viromic analysis. All the protocols of shrimp handling and sampling were approved by the Animal Care and Ethics Committee, Yellow Sea Fisheries Research Institute, Chinese Academy of Fishery Sciences (approval code: YSFRI-2015007).

### 2.2. Transmission Electron Microscopy (TEM) Analysis

The *P. vannamei* hepatopancreas tissues were first fixed in 2.5% glutaraldehyde in 0.1 M PBS (pH 7.4) for 24 h at 4 °C. Secondly, 1% osmium tetroxide was used for 2 h for further fixing of the sample. The sample was then embedded in Spurr’s resin for preparing ultrathin sections (50 nm). The ultrathin sections and the purified viral particles were stained with uranyl acetate and lead citrate, and examined using a JEOL JEM-1200 electron microscope (Jeol Solutions for Innovation, Peabody, MA, USA).

### 2.3. Virus Purification

Purification of viral particles was conducted according to a previously reported protocol with a minor revision [37]. The samples of shrimp were homogenized in TN buffer (20 mM Tris/HCl, 400 mM NaCl, pH = 7.4) and clarified at 1400× *g* for 15 min to gather the supernatant. The pellets were homogenized again in 10 mL TN buffer and centrifuged at 8000× *g* for 15 min at 4 °C. The supernatant was clarified for a second time at 10,000× *g* for 25 min. The final supernatant was then centrifuged at 120,000× *g* for 5 h (Ultracentrifuge CP100WX; Hitachi, Tokyo, Japan). TN buffer was used to suspend the pellet.

### 2.4. RNA Extraction, Library Construction and Virome Sequencing

Total RNA of the viral extracts was prepared by using Trizol reagent kit (Invitrogen, Carlsbad, CA, USA) following the kit’s protocol. The quality of purified RNA was assessed and determined by using Agilent 2100 Bio-analyzer and RNase-free agarose gel electrophoresis, respectively. mRNA was enriched from the total RNA using Ribo-Zero^TM^ Magnetic Kit (Epicentre, Madison, WI, USA). The enriched mRNA was fragmented into short fragments and reverse transcribed into first-strand cDNA. Second-strand cDNA was then synthesized by amplification in vitro. Next, the cDNA fragments were purified, end-repaired, added with poly(A) and ligated to Illumina sequencing adapters. Finally, the second-strand cDNA was size-selected via agarose gel electrophoresis, amplified by PCR, and sequenced.

### 2.5. Assembling and Phylogenetic Analysis

Trinity, a short reads assembling program, was used to finish genome assembling [38]. The sequence of *Pv*PV was submitted for a BLAST (National Center for Biotechnology Information) search, and highly similar matches were included in the dataset for phylogenetic analysis. The complete genome sequence was aligned with Clustal_W as implemented in MEGA 6.0 [39] using the default settings. The alignment file was checked visually for alignment gaps and missing data. A phylogenetic tree was then reconstructed by the neighbor-joining method with bootstrap analysis (1000 replicates) using MEGA 6.0.

### 2.6. Viral RNA Extraction and cDNA Synthesis

Total RNA was prepared from the purified virus suspension using Viral Genome RNA Extraction Kit (Tiangen, Beijing, China), and the quality and quantity of the nucleic acids were measured with the NanoDrop 2000c Spectrophotometer. Then, final concentrations of 100–200 ng/µL of the viral-RNA samples were adjusted for cDNA preparation. The primers used for synthesis of the first-strand cDNA are listed in Table 1.

### 2.7. PCR Amplification and Sequencing

PCR amplification was conducted with TaKaRa EX Taq DNA polymerase (TaKaRa), cDNA template, dNTP and primers following the manufacturer’s protocol. The PCR was performed at 98 °C, with pre-denaturation for 10 s, followed by 35 cycles including 94 °C denaturation for 30 s, annealing for 30 s, extension at 72 °C for 30 s and final extension at 72 °C for 7 min. The annealing temperatures were set based on the requirements of different primers (Table 1). The PCR amplicons were purified and then sequenced by the commercial sequencing company Shanghai SAN-GAN Co. Ltd. (Shanghai, China).

### 2.8. Mass Spectrometry Analysis

To identify the suspected protein(s) of potential viruses, SDS-PAGE (sodium dodecyl sulphate–polyacrylamide gel electrophoresis) of the purified viruses was first conducted, and then mass spectrometry analysis of the potential proteins of the purified virus was carried out. The sample was subjected to enzymatic hydrolysis subsequently according to the previous methods [40,41,42]. The digested samples were then analyzed through mass spectrometry analysis by using the Easy-nLC 1200 (Thermo Scientific, Waltham, MA, USA, P/N LC140) and Q-Exactive HF-X (Thermo Scientific). After the mass spectrum data were extracted using Proteome Discover software, the MaxQuant 1.6.1.0 was used to search the database. The search parameters were as follows: the database was the protein library derived from genomes; in trypsin digestion, the maximum missed cut was 2; the main search of precursor tolerance was 4.5 ppm; the first search of precursor tolerance was 20 ppm; methionine (M) oxidation and asparagine (N) deamidation were set as variable modifications as described in previous research [43].

## 3. Results

### 3.1. TEM Analysis of the Potential Virus and Morphology of the Purified Virions

**Figure 1:**
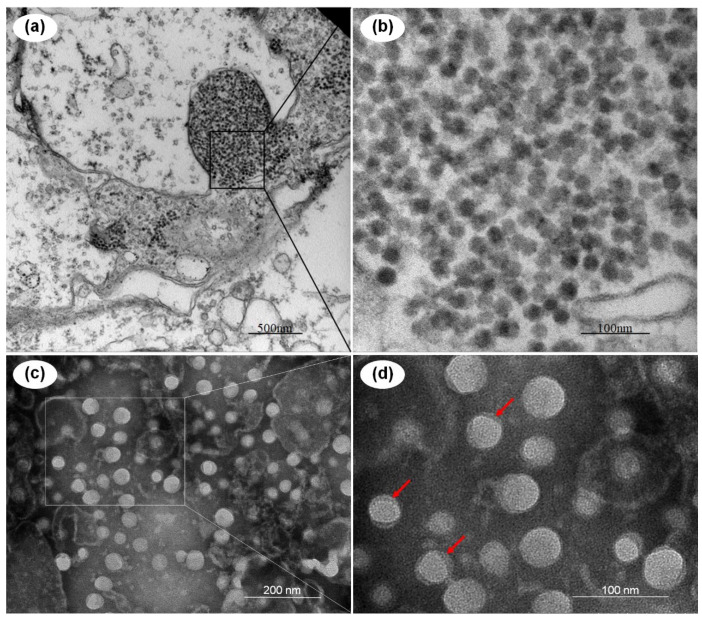
Transmission electron micrographs of the *Penaeus vannamei* picornavirus (*Pv*PV) virions and an ultrathin section of the hepatopancreatic epithelial cells of the moribund *Penaeus vannamei* from the farm in Shandong, China. (**a**,**b**) Ultrathin section of hepatopancreatic epithelial cells; (**c**,**d**) purified *Pv*PV virions; (**b**–**d**) show magnified micrographs in the corresponding framed areas of (**a**–**c**), respectively. Note the tegument of *Pv*PV particles, indicated with red arrows. Scale bars: (**a**) 500 nm, (**b**) 100 nm, (**c**) 200 nm, (**d**) 100 nm.

TEM was used to examine the ultrathin sections of the hepatopancreas tissues of the moribund *P. vannamei*. Un-enveloped virus-like particles with a diameter of ~40 nm (n = 31) were observed in the inclusions body in cytoplasm of hepatopancreatic epithelial cells (Figure 1a). Observation of the negative-stained virus particles purified from the moribund *P. vannamei* by ultracentrifugation revealed that the purified viral particles were identical in morphology with those virus-like particles in ultrathin sections of the hepatopancreas tissues (Figure 1b). The virus was tentatively named *Penaeus vannamei* picornavirus (*Pv*PV) for further investigation.

### 3.2. Molecular Characterization of PvPV

**Figure 2:**
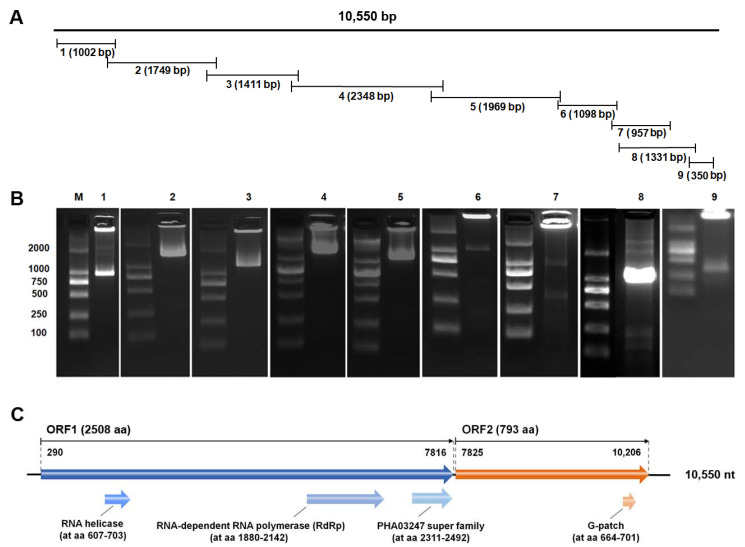
Assembly strategy and agarose gel electrophoresis of reverse transcription PCR (RT-PCR) products of *Penaeus vannamei* picornavirus (*Pv*PV) genome sequence, and schematic presentation of the genomic open reading frame segments and conserved domains of *Pv*PV. (**A**) Strategy of re-cloning and assembling the genomic open reading frame of *Pv*PV by RT-PCR. The numbers 1–9 corresponding to lanes 1–9 in Figure (**B**) represent the expected size of the RT-PCR amplification products and indicate the position of amplicons in the *Pv*PV genome. (**B**) Agarose gel electrophoresis of RT-PCR products for re-cloning of the *Pv*PV genomic sequence. Lane M: 2000 bp marker; lanes 1–9: amplicons of *Pv*PV genomic sequence. (**C**) The potential protein encoding by segments of *Pv*PV genome was identified by using open reading frame (ORF), and the conserved protein domains were predicted using the conserved domain database (CDD) searching tool from the National Center for Biotechnology Information website.

Using Illumina sequencing, the potential pathogens’ sequences were identified from the total RNA of the viral extracts prepared from the diseased *P. vannamei* sample NO. 20150715007. A total of 37,073,944 raw reads were obtained, and then 35,246,406 clean reads were kept after quality control. These reads were de novo assembled using Trinity, and virus-related contigs were submitted to BlastX and BlastN for primary confirmation of potential viral genomes. One potential virus with the most contigs with a whole-genome sequence of 10,550 nts in length was found, and the virus was 96% identical in genomic sequence level with the previous reported Wenzhou shrimp virus 8 (GenBank Number: KX883984.1). To confirm whole sequences of *Pv*PV, primers for Sanger sequencing were designed based on the known gene sequences (Table 1). Amplification of the *Pv*PV genome by using the designed primers, as well as 5′ and 3′ RACE methods, were performed to notarize the *Pv*PV genome. Reconstruction of the sequences from Sanger sequencing of the amplicons (Figure 2A) indicated that the re-cloned *Pv*PV genome was consistent with those from RNA virome sequencing (Figure 2B).

Analysis of the genomic structure indicated that *Pv*PV coded two open reading frames (ORFs). ORF1 contained three deduced conserved domains (Figure 2C), including RNA-dependent RNA polymerase (RdRp domain, cd01699, nt 1880–2142, E-value 1.89 × 10^−39^), RNA helicase (pfam00910, nt 607–703, E-value 7.91× 10^−15^) and PHA03247 super family (large tegument protein UL36, cl33720, nt 2, 311-2, 492, 2.19× 10^−5^). The deduced conserved domain of ORF2 was G-patch (G-patch domain, pfam01585, nt 664-701, E-value 7.98× 10^−8^). The ORF1 RNA segment (nt 290-7, 816) encoded a RdRp, RNA helicase and the large tegument protein UL36 with a total length of 2508 aa. The ORF2 RNA segment (nt 7, 825-10, 206) encoded a G-patch protein with a total length of 793 aa.

### 3.3. Identification of PvPV by Mass Spectrometry Analysis

**Figure 3:**
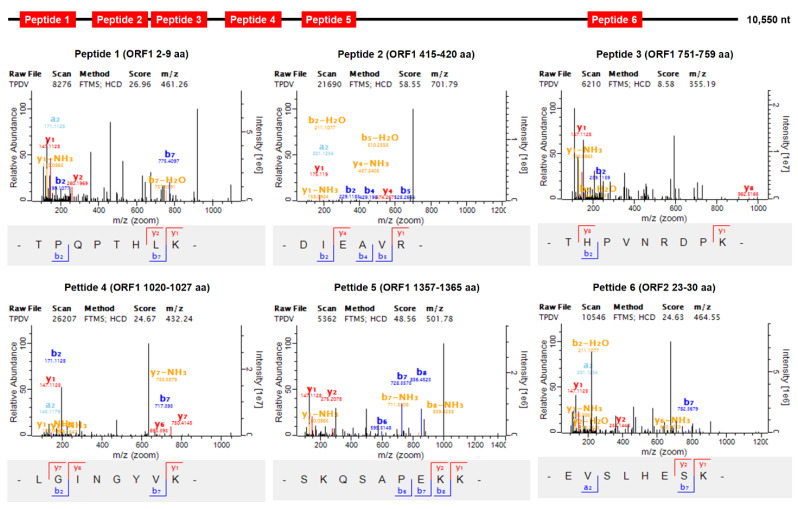
Mass spectrometry analysis of *Penaeus vannamei* picornavirus (*Pv*PV) proteins. To identify *Pv*PV protein by matrix-assisted laser desorption ionization time-of-flight mass spectrometry analysis, six peptides were screened and measured in the digested *Pv*PV protein using trypsin.

To identify the purified viral particles isolated from the diseased *P. vannamei* sample NO. 20150715007, SDS-PAGE and mass spectrometry analyses were carried out (Supplementary Figure S1). The major bands of potential viral proteins in the SDS-PAGE electrophoresis were excised from the gel and identified using mass spectrometry. The results of mass spectrometric analysis showed that the sequences of the identified peptides matched exactly with the deduced amino acids encoding by the open reading frame sequence of the *Pv*PV genome (Figure 3). That is, mass spectrometry analysis further revealed the presence of *Pv*PV in the diseased shrimp individuals.

### 3.4. Phylogenetic Analysis

**Figure 4:**
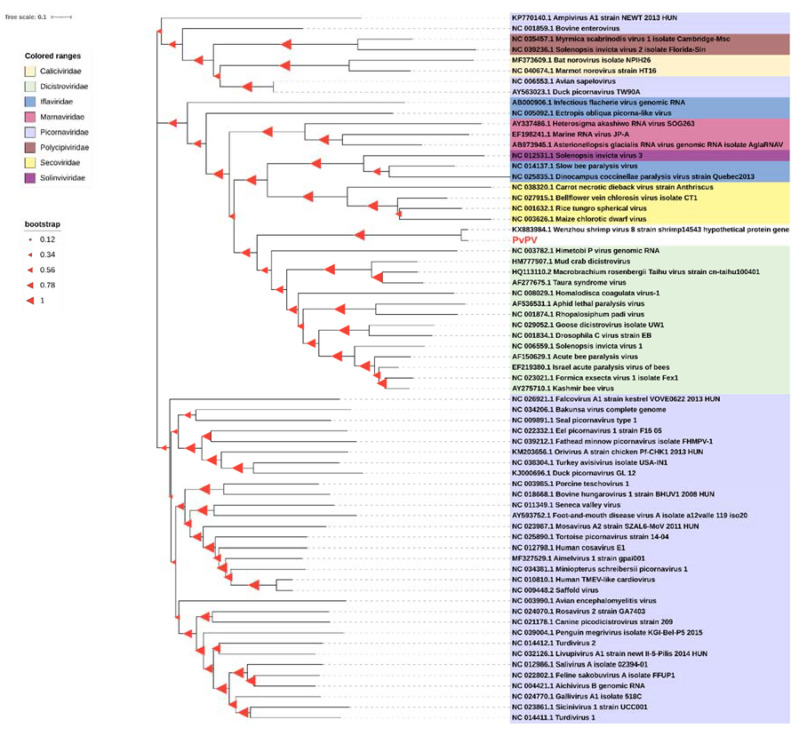
Phylogeny analysis of the *Penaeus vannamei* picornavirus (*Pv*PV) and other picornaviruses. Phylogenetic trees based on the full-length genomic sequence were constructed using the neighbor-joining method with 1000 bootstrap replicates under the parameter of complete deletion and Poisson model using the software MEGA 6.0. The size of the red triangle symbol at the branch nodes represents the bootstrap confidence levels of the 1000 bootstrap replications obtained. Scale bar was 0.1. The *Pv*PV is highlighted in bold red.

To determine the phylogenetic relationship of the virus found in sample NO. 20150715007 with other picornaviruses, phylogenetic analyses were performed based on the genome sequences both from *Pv*PV and the corresponding picornaviruses in the order *Picornavirales* (Figure 4). In the tree, the picornaviruses used in the multiple alignments were subdivided into eight identified families: *Caliciviridae*, *Dicistroviridae, Iflaviridae*, *Marnaviridae*, *Picornaviridae*, *Polycipiviridae*, *Secoviridae* and *Solinviviridae*. The potentially new picornavirus isolated from shrimp sample NO. 20150715007 was located in a new branch that belongs to the *Dicistroviridae* family and *Picornavirales* order. In the new branch, *Pv*PV clustered together with Wenzhou shrimp virus 8 (GenBank: KX883984.1), sharing a high sequence identity of 95.43%. These results of phylogenetic analysis of genome sequence indicated *Pv*PV was a close relative of Wenzhou shrimp virus 8. In a more refined evolutionary tree based on the most related viral genomes, *PvPV* seems to be classified into the genus *Aparavirus* (Supplementary Figure S2). However, according to the new phylogenetic tree based on the deduced amino acids of RdRp genes from the most related viruses, both *Pv*PV and Wenzhou shrimp virus 8 may be classified into a completely new evolutionary group, which is markedly different from the family *Dicistroviridae* (Supplementary Figure S3).

## 4. Discussion

Over the past decade, the world shrimp farming industry has been heavily affected by several emerging and re-emerging diseases [2,44,45]. Among the infectious diseases of cultured shrimp, certain virus-caused diseases stand out as the most significant [46]. Thus, early investigation and identification of the potential viral pathogens is very important for the prevention and control of shrimp emerging diseases. In our research, based on preliminary epidemiological investigation by our laboratory, we reported a novel picornavirus discovered in moribund oriental shrimp *P*. *vannamei* farmed in China. This novel shrimp virus was identified following study of virus morphology, viral gene sequencing, viral mass spectrometry analysis and phylogenetic analysis. The investigation results show that the genomic sequence of *Pv*PV, sharing high similarity with that of Wenzhou shrimp virus 8, represents a new species.

*Picornavirales* is a proposed order of positive-sense single-stranded RNA viruses with a pseudo-T = 3 virion architecture [24]. The order *Picornavirales* consists of eight families: *Caliciviridae*, *Dicistroviridae*, *Iflaviridae*, *Marnaviridae*, *Picornaviridae*, *Polycipiviridae*, *Secoviridae* and *Solinviviridae* [24,47]. Based on genome sequencing and phylogenetic analysis, *Pv*PV proved to belong to the family *Dicistroviridae* in our study. The family *Dicistroviridae* mainly contains three genera: *Aparavirus* (type species: *Acute bee paralysis* virus), *Cripavirus* (type species: *Cricket paralysis* virus) and *Triatovirus* (type species: *Triatoma* virus) [7]. There are also some tentative members of the family *Dicistroviridae*. Currently, only three species of dicistroviruses have been reported in crustaceans. One was TSV in *P*. *vannamei* [7,32,48,49], as an agent of TSV epizootics. Cumulative mortalities due to TSV epizootics have ranged from 40% to >90% in cultured populations of postlarval (PL), juvenile and subadult *P*. *vannamei* [50]. One was mud crab dicistrovirus-1 (MCDV-1), an infection which caused 100% mortality in crabs [51]. The other was *Macrobrachium rosenbergii* Taihu virus (MrTV) in crustaceans, which was confirmed to be the causative agent of the larval mortality syndrome in *M. rosenbergii* [52], causing a high mortality rate of up to 80–90% [53]. The high mortality in crustaceans caused by the three species of dicistroviruses results in economic loss in aquaculture of crustaceans. This study reported a fourth dicistrovirus, *Pv*PV, in crustaceans, having a close relationship with the Wenzhou shrimp virus 8 (WzSV-8), MCDV-1, MrTV and TSV as shown by phylogenetic analysis. However, the potential pathogenicity of the virus is still unclear and deserves further study.

*Pv*PV and WzSV-8 have previously been classified into the genus *Aparavirus* in the family *Dicistroviridae* according to the evolutionary tree based on viral genomes. However, according to the phylogenetic tree based on the deduced amino acids of RdRp genes from the most related viruses, *PvPV* and WzSV-8 may be re-classified into a completely new evolutionary group markedly different from the family *Dicistroviridae*. In this study, compared with the RdRp and whole genomes of known viruses, the RdRp of *Pv*PV shared higher similarity (highest at 29.79% for a matching pair with Feksystermes virus) than did the genome of *Pv*PV (highest no more than 10% matching pair with any known viruses). The result showed that the deduced amino acids sequences of RdRp were more conservative than the genome sequence in *Pv*PV. Therefore, it can be deduced that the result of phylogenetic analysis based on RdRp would be more reliable than that based on the genome in *Pv*PV when carrying out more refined genetic phylogeny analysis. The finding that *Pv*PV may be categorized as a completely new evolutionary group markedly different from the family *Dicistroviridae* is thus more reliable and credible. Hence, *PvPV* should be a new species that also represents a newly proposed genus in the *Picornavirales* order. This proposal will be submitted to the International Committee on Taxonomy of Viruses (ICTV) for approval.

In dicistrovirus, the RNA genome is monopartite and dicistronic with two non-overlapping ORFs separated and flanked by the untranslated regions (UTRs) [52]. In TSV, ORF 1 contains the sequence motifs for nonstructural proteins, such as helicase, protease and RdRp. ORF 2 contains the sequences for TSV structural proteins, including the three major capsid proteins VP1, VP2 and VP3 [46]. In MrTV, the components of ORF 1 contain an RNA helicase (Hel), a RdRp and a cysteine protease. The ORF2 encodes the capsid protein, 990 amino acids in length [52]. In MCDV-1 genome, three conserved domains, including BIR (Baculovirus Inhibitor of apoptosis protein Repeat), helicase and RdRp were found with BLAST in the National Center for Biotechnology Information (NCBI) website. ORF2 of MCDV-1 encoded the capsid proteins VP1, VP2 and VP3 with 961 putative amino acids [51]. Genomic structural analysis revealed that the *Pv*PV genome was arranged in this typical organization. In *Pv*PV, two separated ORFs, flanked by UTRs, were identified. The 5′-proximal ORF1 encodes RdRp, helicase and UL36 (large tegument protein), while the 3′-proximal ORF2 was deduced to encode G-patch. In all +ssRNA viruses, the *RdRp* gene is the most conserved gene. Evolution of RNA viruses is defined and clarified mostly based on RdRp phylogeny supplemented by phylogenies of other genes that are conserved in subsets of viruses, together with comparative analysis of genome organization [54]. RNA helicase A (RHA) has been shown to play a proviral role in the life cycle of many viruses [55]. All herpesviruses have a tegument, a layer of protein located between the virus capsid and membrane. Large tegument protein UL36 plays a central role in organizing the overall structure of the tegument [56]. In the pseudorabies virus (PrV), pUL36 is the only tegument protein analyzed to date, which is strictly essential for viral replication [57]. G-patch proteins, consisting of glycine-rich motifs, act as cofactors in RNA processing [58,59,60]. Similar to the genome structure of other dicistroviruses in crustaceans, the ORF1 of *Pv*PV also includes RdRp and helicase. The ORF1 of *Pv*PV does not include proteases, a cysteine protease, or BIR but includes the large tegument protein UL36. At the same time, a conserved domain database (CDD) search identified homologous proteins to the G-patch protein in ORF2, rather than the capsid protein, which is different from most described dicistroviruses. The existence of a G-patch protein instead of the capsid protein expands our awareness of the structural flexibility and diversity among dicistroviruses. The difference in genome sequence between *Pv*PV and other dicistroviruses supports the view that *Pv*PV belongs to a new genus. Genomic structural analysis lays the foundation for further exploration of the pathogenic mechanism of *Pv*PV.

In conclusion, *Pv*PV, a novel picornavirus in moribund white leg shrimp *P. vannamei*, was discovered and characterized as a novel member of the *Dicistroviridae* based on virus morphology, viral genome, viral mass spectrometry analysis and phylogenetic analysis. The discovery of *Pv*PV expands the family of *Dicistroviridae* and may shed light on potential *Pv*PV disease prevention and occurrence.

## Figures and Tables

**Table 1:** Primer used for confirmation of the whole genome of *Penaeus vannamei* picornavirus.

Gene Fragments	Primer Code	PCR Annealing Temperature	Primer Sequence (5′-3′)	Size (bp)
1	RNA-F1	48 °C	ATCCACGGAAAGAGCC	1002 bp
	RNA-R1		TAGCGGAATGCGACAA	
2	RNA-F2	45 °C	CTGCCCTTTGCCGTCTTC	1749 bp
	RNA-R2		CTGAGTGTCATTGTCTTGGA	
3	RNA-F3	50 °C	CGTTCCCATAAGGACCCA	1411 bp
	RNA-R3		ATATCGCTTTCCAGAGGC	
4	RNA-F4	50 °C	CTCAGTCGTCTCCCGTGTC	2348 bp
	RNA-R4		CGGTCTCAAAGTCAATCCC	
5	RNA-F5	50 °C	GACGAGTTGAGCCTACAGA	1969 bp
	RNA-R5		ATGCCTTGGAGGAGTGAA	
6	RNA-F6	44 °C	CCCTTCACTCCTCCAA	1098 bp
	RNA-R6		GAGTAATCCTGACATCCC	
7	RNA-F7	40 °C	TACGACCGTAACAATG	957 bp
	RNA-R7		GGCTGAGGAGGAGGAG	
8	RNA-F8	39 °C	CTCTCATACTGCACCA	1331 bp
	RNA-R8		AAATTGCAGGGATTAAATTG	
9	RNA-F9	44 °C	ACGGTGAAGTGAACGC	350 bp
	RNA-R9		TTTTCTCAAAAAGTGTGG	

## Data Availability

The full-length genome of *Pv*PV has been deposited in GenBank under accession number: OK662577 (10,550 nts).

## Supplementary Materials

The following are available online at https://www.mdpi.com/article/10.3390/v13122381/s1, Figure S1: Identifying *Pv*PV proteins by SDS-PAGE (6–8% gel). Lane M, molecular weight markers. Lane 1, the viral structural proteins of *Pv*PV. Figure S2: Phylogeny analysis of the genome sequences of *Pv*PV and representative viruses in the family *Dicistroviridae*. The scale bar was 0.1. Figure S3: Phylogeny analysis of the deduced amino acid sequences of RNA-dependent RNA polymerase (RdRp) gene of *Pv*PV and representative viruses in the order *Picornavirales*. The scale bar was 0.2.
