# Supplementary material for: A Novel Picornavirus Discovered in White Leg Shrimp Penaeus vannamei
Source: Viruses. 2021 Nov 28;13(12):2381. doi: 10.3390/v13122381 (PMC8706678; doi:10.3390/v13122381)
Supplement: Supplementary file 1 [file viruses-13-02381-s001.zip › viruses-1454172-supplementary.pdf]

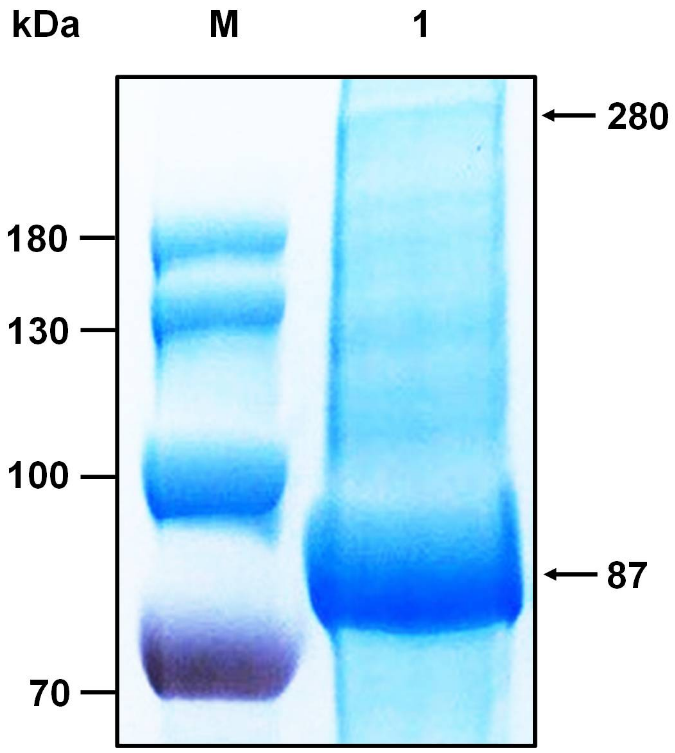

Figure S1. Identifying *Pv*PV proteins by SDS-PAGE (6-8% gel). Lane M, molecular weight markers. Lane 1, the viral structural proteins of *Pv*PV.

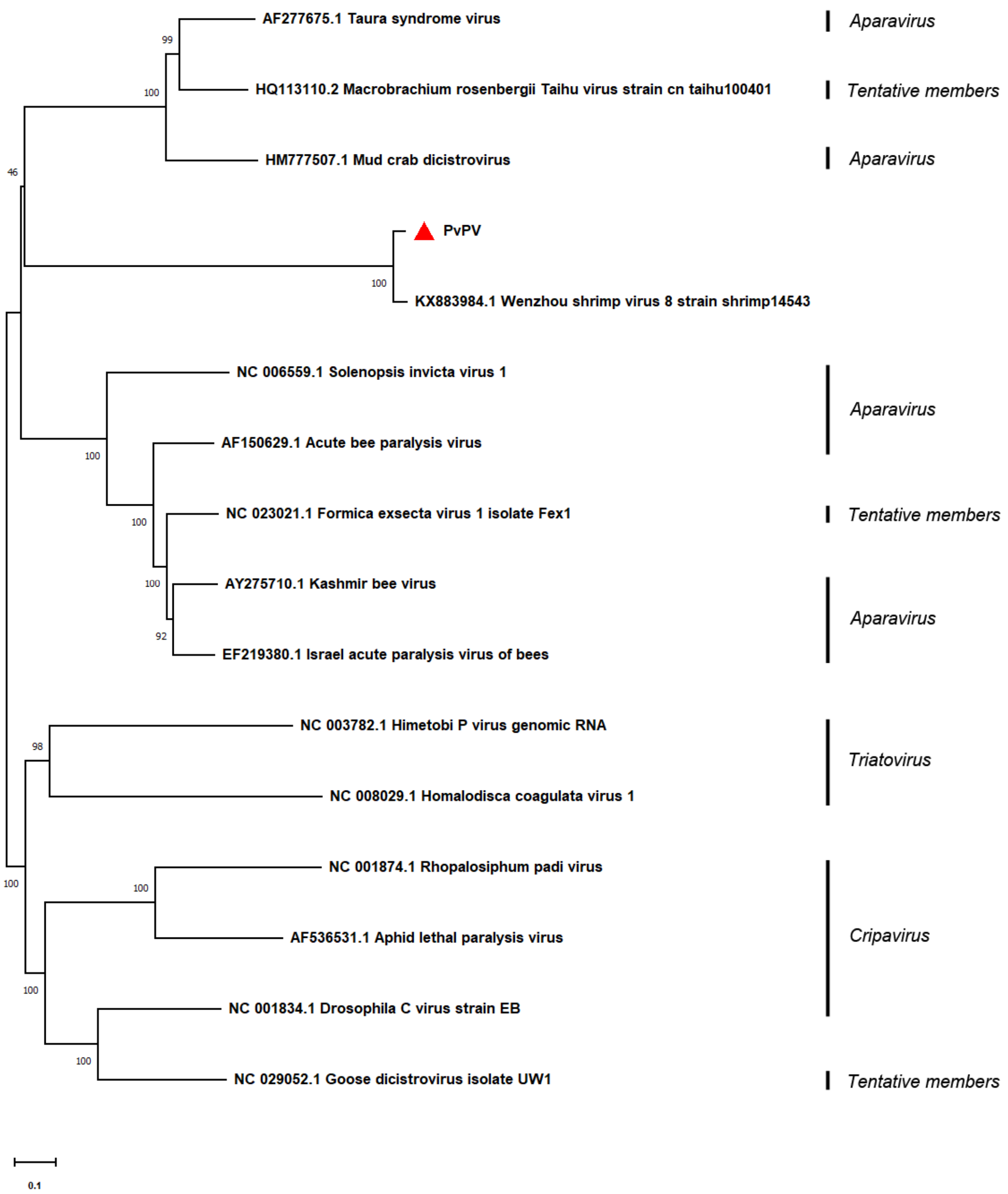

Figure S2. Phylogeny analysis of the genome sequences of *Pv*PV and representative viruses in the family *Dicistroviridae*. The scale bar was 0.1.

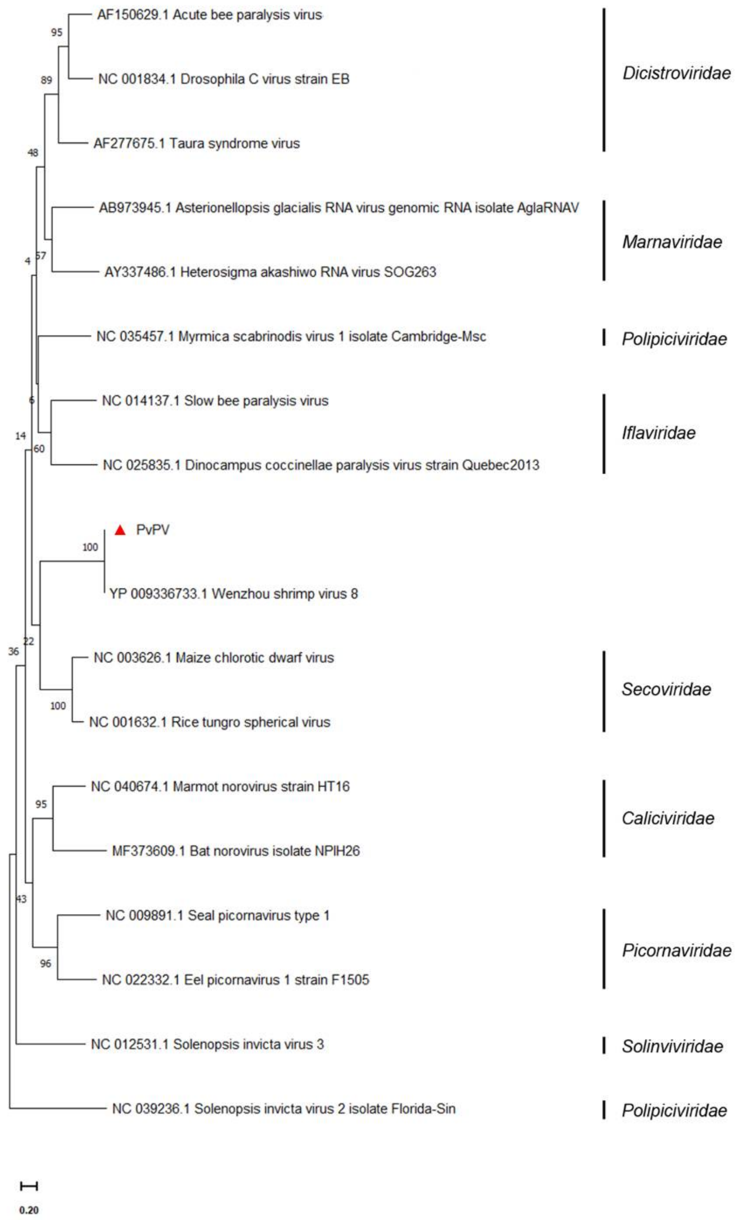

Figure S3. Phylogeny analysis of the deduced amino acid sequences of RNA-dependent RNA polymerase (RdRp) gene of *Pv*PV and representative viruses in the order *Picornavirales*. The scale bar was 0.2.
